# Comparison between modified and conventional combined Trabeculotomy-Trabeculectomy with Mitomycin-C for intraocular pressure reduction in primary congenital glaucoma

**DOI:** 10.12669/pjms.40.9.8788

**Published:** 2024-10

**Authors:** Ahmed Raza, Muhammad Ajmal Chaudhary, Asma Mushtaq, Asad Aslam Khan

**Affiliations:** 1 Ahmed Raza, MBBS, FCPS, FPO Department of Pediatric Ophthalmology, The University of Child Health Sciences, Lahore, Pakistan; 2 Muhammad Ajmal Chaudhary, MBBS, DOMS, MCPS, FCPS, FPO Department of Pediatric Ophthalmology, Sheikh Zayed Medical College, Rahim Yar Khan, Pakistan; 3 Asma Mushtaq, MBBS, FCPS, FRCS, FPO Department of Pediatric Ophthalmology, The University of Child Health Sciences, Lahore, Pakistan; 4 Asad Aslam Khan. MBBS, MS Department of Ophthalmology, COAVS/KEMU, Lahore, Pakistan

**Keywords:** Primary congenital glaucoma, Trabeculotomy, Trabeculectomy, Combined trabeculotomy– trabeculectomy and Pediatric glaucoma surgery

## Abstract

**Objectives:**

To determine the efficacy of modified combined trabeculotomy-trabeculectomy (CTT) with mitomycin-C (MMC) versus conventional CTT with MMC for intraocular pressure (IOP) reduction in patients with primary congenital glaucoma (PCG).

**Methods:**

This interventional study was carried out in The Institute of Ophthalmology, Lahore from January 2018 to June 2019. We included 70 patients of either gender, with the age range from birth to ten years and with PCG having IOP >30 mm Hg. These patients were divided in groups A and B with 35 patients in each group. Patients in Group-A underwent modified CTT with MMC and in Group-B conventional CTT with MMC was carried out.

**Results:**

The mean age of our patients was 15.17 ± 12.39 months. Regarding gender, 57 (81.4%) were males and 13 (18.6%) were females. Baseline mean IOP in Group-A was 32.49 ± 1.01 mm Hg whereas in Group-B mean IOP was 31.97 ± 1.04 mm Hg. After one month, the mean IOP was 27.60 ± 1.72 mm Hg in Group-A, whereas mean IOP was 27.40 ± 1.82 mmHg in Group-B. After three months, the mean IOP was 21.63 ± 1.09 mm Hg in Group-A, whereas 20.97 ± 1.12 mm Hg in Group-B. In Group-A efficacy was achieved in 14 (40%) patients whereas in Group-B efficacy was achieved in 25 (71.4%) patients.

**Conclusion:**

Our study revealed that conventional CTT with MMC showed better efficacy than modified CTT with MMC for reduction of IOP in PCG.

## INTRODUCTION

Congenital glaucoma is a major cause of blindness in the pediatric age group. It is characterized by elevated intraocular pressure (IOP) and subsequent damage to the optic nerve. The incidence in the western countries lies within 1:10,000 to 1:70,000 births.^1^ The term primary congenital glaucoma (PCG) is used when it occurs between the time of birth and three years of age in the absence of any secondary cause or related systemic problems. Children with infantile PCG might have an early onset at neonatal age or a late onset at >01-24 months of age.^1^ PCG eyes have an isolated trabeculodysgenesis, about 75% cases are bilateral and about 65% cases are male. Most of the PCG cases are sporadic with no evidence of hereditary pattern. Although a hereditary pattern is found in about 10% of cases, which is considered to be an autosomal recessive trait. Most researchers believe polygenic^2^ pattern of inheritance in PCG patients.

Usually, parents notice a classical triad of symptoms i.e., photophobia, tearing and blepharospasm in bright lights.^3^ Corneal haze/enlargement, Haab’s striae, buphthalmos^4^, and in severe cases lens dislocation^4^ and globe perforation is seen. Raised IOP is the hallmark of PCG. Enlarged cup-disc ratio (CDR)^5^ of > 0.2 and/or asymmetric cupping are suspicious of PCG. Increased horizontal corneal diameter (HCD), above 11mm in first year of life^5^ and above 12-13mm thereafter are highly suggestive of PCG.

Medical therapy is required to reduce IOP and to get corneal clarity prior to surgical treatment.^6^ Essential treatments in PCG is surgical, aimed to abolish the resistance to aqueous outflow^7^ with the goal to control IOP permanently and preserving visual function. Goniotomy and trabeculotomy with high reported success rates are considered first line treatments of choice in PCG management. Combined trabeculotomy-trabeculectomy (CTT) is the most preferred procedure in our circumstances because many patients have very late presentation with advance disease. MMC is a chemotherapeutic agent, used during filtration surgery to increase the success rate; usually 0.4 mg/ml for 02 minutes is used.^6^ The success rate of angle surgery is about 33-80% after 01-03 procedures.^8^ Recently, tissue engineered biodegradable implants like Ologen are used to enhance the success rate of trabeculectomy.^9^

Several surgical procedures with potential risks and benefits are available for PCG. The optimal first-line surgery and whether CTT is superior to trabeculotomy alone are debatable. Recent studies have found that CTT has superior results as compared to conventional procedures.^7^ Our study will try to answer; is there an efficacy of modified CTT with MMC in the reduction of IOP in PCG when it is compared to conventional CTT with MMC? Our hypothesis was that modified CTT with MMC is more efficacious as compare to conventional CTT with MMC. Our study will provide our experience of CTT with MMC for PCG in our population.

## METHODS

This interventional study was carried out in The Institute of Ophthalmology, Unit-III, Mayo Hospital, Lahore, from January 2018 to June 2019. We studied 70 patients; these were divided in two groups A and B with 35 patients in each group. Sample size was estimated using non-probability consecutive sampling technique by using 05% level of significance, 90% power of test with expected % age in Group-A as 100%^10^ and in Group-B as 79%.^10^

### Ethical Approval:

This proposal was approved from institutional review board (No. 252/RC/KEMU, Date: 17-11-2017).

Patients of either gender with the age range from birth to ten years, presented with PCG having IOP >30 mm Hg were included while patients with borderline IOP of 21-30 mm Hg, patients having glaucoma associated with other congenital or acquired abnormalities and patients with previous history of intraocular surgery were excluded. Before taking the informed consent, whole procedure was explained to the parents of PCG children.

Ophthalmic and systemic history was taken in detail and through ophthalmic examination was carried out. When needed B-scan was done to rule out any posterior segment pathology. Demographic details like name, age, gender, anatomical site were recorded. After initial examination of patients in outpatient department (OPD), diagnosis was established by doing examination under anesthesia (EUA) in the operation theater. HCD was measured with Vernier Caliper, IOP was measured by using hand held Perkin’s applanation tonometer and CDR examination was carried out with indirect ophthalmoscope. Where possible pupil examination, refraction, axial length measurement and corneal photos were taken.

In Group-A, modified CTT with MMC while in Group-B, conventional CTT with MMC were carried out under general anesthesia (GA). Tobramycin 0.3%, dexamethasone 0.1% and Cyclopentolate 01% eye drops were given postoperatively to all patients. After surgery, all patients were examined in the eye ward for red reflex, eye injection, corneal edema and anterior chamber formation for few days. Follow-up EUA to check IOP, HCD, corneal clarity, bleb status, fundus examination and other postoperative complications was done at one week; and at one, two and three months. Efficacy^8^ was labeled when IOP ≤ 21 mm Hg was noted. All information was recorded in a designed performa.

### Statistical Analysis:

Data analysis was done by special package for social sciences (SPSS) version-21. Quantitative variables like age, IOP were presented as mean and standard deviation (SD) while qualitative variables like gender, anatomical site and efficacy were presented as frequencies and percentages. Efficacy of both the groups was compared by using chi-square test. P-value of ≤ 0.05 was taken as significant.

## RESULTS

In this study total 70 patients were enrolled. The mean age of our patients was 15.17 ± 12.39 months with minimum and maximum ages of two and 48 months respectively. Mean age of Group-A patients was 15.14 ± 11.89 months whereas in Group-B mean age was 15.20 ± 13.06 months. According to our study, 57 (81.4%) were males whereas 13 (18.6%) were females, male to female ratio was 4.4: 1.0. In Group-A, there were 30 (85.7%) males and 05 (14.3%) were females. In Group-B, there were 27 (77.1%) males and 08 (22.9%) were females. Our study showed that in Group-A, left anatomical site was noted in 17 (45.9%) patients whereas right anatomical site was noted in 18 (54.5%) patients. In Group-B, left anatomical site was noted in 20 (54.1%) patients whereas right anatomical site was noted in 15 (45.5%) patients.

At baseline, the mean IOP in Group-A was 32.49 ± 1.01 mm Hg whereas in Group-B mean IOP was 31.97 ± 1.04 mm Hg. Statistically significant difference found between both groups at baseline i.e., p-value = 0.040 as shown in Table-I.

**Table-I:** Comparison of baseline IOP.

		Study Groups	

		Group-A	Group-B
Baseline IOP (mm Hg)	n	35	35
	Mean	32.49	31.97
	SD	± 1.01	± 1.04

Independent t-test: -2.095, p-value: 0.040, Group-A: Modified CTT with MMC, Group-B: Conventional CTT with MMC.

After one month, the mean IOP was 27.60 ± 1.72 mm Hg in Group-A, whereas mean IOP was 27.40 ± 1.82 mmHg in Group-B. Statistically insignificant difference found between both groups i.e., p-value = 0.638. After two months, the mean IOP was 24.17 ± 0.89 mm Hg in Group-A, whereas 24.37 ± 1.03 mm Hg in Group-B. Statistically insignificant difference found between both groups i.e., p-value = 0.338. After three months, the mean IOP was 21.63 ± 1.09 mm Hg in Group-A, whereas 20.97 ± 1.12 mm Hg in Group-B. Statistically significant difference found between both groups i.e., p-value = 0.015 as shown in Table-II.

**Table-II:** Comparison of IOP at 3^rd^ month.

		Study Groups	

		Group-A	Group-B
IOP at 3^rd^ month	n	35	35
	Mean	21.63	20.97
	SD	± 1.09	± 1.12

Independent t-test: -2.486, p-value: 0.015, Group-A: Modified CTT with MMC, Group-B: Conventional CTT with MMC.

According to our study the efficacy was achieved in 39 (55.7%) patients while in 31 (44.3%) patients efficacy (IOP ≤ 21mmHg) could not be achieved. In Group-A, the efficacy was achieved in 14 (40%) patients whereas in Group-B the efficacy was achieved in 25 (71.4%) patients. The difference was statistically significant i.e., p-value = 0.008 as shown in Table-III.

**Table-III:** Comparison of efficacy.

		Study Groups		Total

		Group-A	Group-B	
Efficacy	Yes	14 (40%)	25 (71.4%)	39 (55.7%)
	No	21 (60%)	10 (28.6%)	31 (44.3%)
Total		35 (100.0%)	35 (100%)	70 (100.0%)

Chi-square value: 7.006, p-value: 0.008, Group-A: Modified CTT with MMC, Group-B: Conventional CTT with MMC.

## DISCUSSION

Worldwide, PCG is a major cause of childhood blindness. PCG is a challenging disease, requires perfect timing, an appropriate surgical technique and lifetime follow-up.^11^ Unfortunately, PCG may remain undiagnosed for many years and child with “big beautiful eyes” gradually loses his vision.^12^ Delayed diagnosis and treatment of PCG in these children may lead to the optic nerve damage.^13^ Surgical treatment protects and saves visual function^14^ by reducing IOP effectively. Usual treatment options for PCG are goniotomy and trabeculotomy. Trabeculectomy^15^,^16^ is a next surgical choice after failure of goniotomy and trabeculotomy. Surgical intervention in buphthalmic eyes having disturbed anatomy is always a challenge with higher failure rate and multiple complications as compared to adults.^17^

Incidence of PCG in pediatric population of Pakistan is nine times higher as compared to Caucasian population.^18^ GLC3A involvement in the Pakistani population is higher and might be attributable to the mutations in CYP1B1.^18^ Problems in the management of PCG in Pakistan include poor socioeconomic status, delayed treatment acquisition and poor follow-up compliance.^19^ In Pakistan, few studies have been carried out regarding surgical intervention in PCG patients. Qayyum A et al.^20^ in their study in Pakistan, reported average IOP reduction from 30 to 12 mm of Hg in PCG patients following trabeculotomy, follow up period was up to eight months. In a study conducted in PCG patients by Shakir M et al.^19^ in Pakistan, the success of CTT was achieved in 80% cases with IOP reduction from 32 to 13 mm of Hg, mean duration of follow-up was 8.25 months. Shoaib et al.^21^ from Pakistan in their study showed success in 21 (88%) of cases after CTT with MMC with three months follow up. Outcome measure of success in these studies was significant reduction of post-operative IOP i.e., 15 to 21 mm of Hg.

Mandal et al. in a study from India, showed sfimilar success rate but MMC was not used in primary surgery. They concluded that CTT is an effective and safe treatment in advanced PCG patients having corneal diameter of 14 mm or more. In another study of primary CTT, Mandal et al. reported prolonged control of IOP in these patients and normal VA was gained in 42% of the patients.^3^,^6^

A study in Ghanaian children with PCG conducted by Essuman VA et al.^7^ showed that the overall success of CTT was 79%. While another study by Wu Z-K et al.^10^ showed that IOP control was achieved in 100% patients with modified CTT with MMC. Mullaney PB et al.^22^ reported 78% operative success (IOP, ≤ 21 mm Hg) in uncomplicated PCG while Al-Hazmi A et al.^23^ reported 80% success rate in moderate PCG and 70% success rate in severe PCG. Both of these studies used MMC in primary CTT.

Sood D et al.^24^ in their study evaluated the long term outcome of CTT. They showed that the mean IOP reduction was 22.71 ± 11.28 mm Hg. Qualified success was achieved in 148 (66.7%) and modified qualified success in 140 (63.1%). Recent study by Khairy MA et al.^17^ showed complete success (IOP ≤21 mmHg) in 52 of the cases (82.5%) of CTT.

In our study, the mean age of our patients was higher than the studies conducted by Mandal et al.^3^, Essuman VA et al.^7^, Mullaney PB et al.^22^, Al-Hazmi A et al.^23^ and Sood D et al.^24^ This difference could be due to poor socioeconomic status of Pakistani patients who do not seek early treatment until their condition is severe enough. In our study, male children were more similar to the above conducted studies.^3^,^22^-^24^ Our study showed that overall efficacy was achieved in 55.7% patients, which is almost comparable to the above published studies^6^,^7^,^22^-^24^ whereas there is some discrepancy from studies.^10^,^17^ This difference could be due to an elder age group, a small sample size and a short follow-up period in our study.

According to our study conventional CTT with MMC group showed better results than modified CTT with MMC group. Currently, a little literature is available regarding surgical management of PCG in Pakistani children. Our study will be an addition to the available national and international research evidence on this important topic.

### Limitations:

It was a single center study with relatively a small sample size and a short follow-up period. So, our results are not a real picture of our pediatric population.

## CONCLUSIONS

Our study has tried to provide an insight in the management of PCG with current evidence. Our study revealed that conventional CTT with MMC showed better efficacy than modified CTT with MMC for the reduction of IOP in PCG patients. Thus, in future, we recommend conventional CTT with MMC as an optimal primary surgical procedure to treat PCG patients in Pakistan and in other developing countries.

### Recommendations:

PCG requires early and proper surgical intervention, good compliance and lifelong follow-up to achieve a favorable outcome with preservation of vision. Further well-designed randomized controlled trials (RCT) with larger sample size and longer follow-up period are required to improve the management of PCG patients.

### Authors Contribution:

**AR:** Did data collection, Statistical analysis and manuscript writing.

**MAC:** Conceived, designed and did editing of manuscript, is responsible for integrity of research.

**AM:** Did data collection, interpretation of data and manuscript writing.

**AAK:** Did review and final approval of manuscript.

## References

[1] Yu Chan JY, Choy BNK, Ng ALK, Shum JWH (2015). Review on the Management of Primary Congenital Glaucoma. J Curr Glaucoma Pract.

[2] Singab AAS, Mohammed OA, Saleem MIH, Abozaid MA (2017). A Comparative Study:The Use of Collagen Implant versus Mitomycin-C in Combined Trabeculotomy &Trabeculectomy for Treatment of Primary Congenital Glaucoma. J Ophthalmol.

[3] Mandal AK, Gothwal VK, Khanna R (2022). Combined trabeculotomy-trabeculectomy for primary congenital glaucoma:long-term experience from a tertiary referral centre in a developing nation. Acta Ophthalmol.

[4] Ho CL, Walton DS (2004). Primary congenital glaucoma:2004 update. J Pediatr Ophthalmol Strabismus.

[5] Fang L, Guo X, Yang Y, Zhang J, Chen X, Zhu Y (2020). Trabeculotomy versus combined trabeculotomy-trabeculectomy for primary congenital glaucoma:study protocol of a randomised controlled trial. BMJ Open.

[6] Mandal AK, Chakrabarti D (2011). Update on congenital glaucoma. Indian J Ophthalmol.

[7] Essuman VA, Braimah IZ, Ndanu TA, Ntim-Amponsah CT (2011). Combined trabeculotomy and trabeculectomy:outcome for primary congenital glaucoma in a West African population. Eye.

[8] Ozawa H, Yamane M, Inoue E, Yoshida-Uemura T, Katagiri S, Yokoi T (2017). Long-term surgical outcome of conventional trabeculotomy for childhood glaucoma. Jpn J Ophthalmol.

[9] Senthil S, Rao HL, Babu JG, Mandal AK, Garudadri CS (2013). Comparison of outcomes of trabeculectomy with mitomycin C vs. ologen implant in primary glaucoma. Indian J Ophthalmol.

[10] Wu ZK, Wu J, Tan Q, Jiang J, Song WT, Xia XB (2016). Therapeutic effect analysis on the treatment of congenital glaucoma through modified combined trabeculotomy-trabeculectomy. Int J Ophthalmol.

[11] Aktas Z, Gulpinar Ikiz GD (2023). Current surgical techniques for the management of pediatric glaucoma:A literature review. Front. Ophthalmol.

[12] Yonekawa Y, VanderVeen DK, Shah AS (2013). Congenital glaucoma. J Pediatr.

[13] Karaconji T, Zagora S, Grigg JR (2022). Approach to childhood glaucoma:a review. Clin Exp Ophthalmol.

[14] Xiahou L, Liu C, Zhou W, Yang S (2020). Microsurgical scleral drainage and trabeculectomy-scleral flap adjustable suture combination technique in the treatment of primary glaucoma. Pak J Med Sci.

[15] Mengal M, Khan MA, Khan A, Ahmed M, Chaudhry RK, Khan NQ (2020). Outcomes of Trabeculectomy in Congenital Glaucoma;Experience in Helpers Eye Hospital Quetta. Pak J Ophthalmol.

[16] Kessel L, Pedersen KB, Siersma V, Kappelgaard P, Bach-Holm D (2021). Long-term success after trabeculotomy in primary congenital glaucoma–a study with up to 35 years follow-up. Acta Ophthalmologica.

[17] Khairy MA, Kenawy S, Fawzi KM, Al-Nashar HY (2022). Factors Affecting Final Surgical Outcome of Combined Trabeculotomy–Trabeculectomy in Primary Congenital Glaucoma. Clin Ophthalmol.

[18] Bashir R, Sanai M, Azeem A, Altaf I, Saleem F, Naz S (2014). Contribution of GLC3A locus to Primary Congenital Glaucoma in Pakistani population. Pak J Med Sci.

[19] Shakir M, Bokhari SA, Kamil Z, Zafar S (2013). Combined trabeculotomy and augmented trabeculectomy in primary congenital glaucoma. J Coll Physicians Surg Pak.

[20] Qayyum A, Baloch RA (2014). Trabeculotomy in Primary Congenital Glaucoma. Pak J Ophthalmol.

[21] Shoaib KK, Anwar S, Jawwad AAM (2022). Combined Trabeculotomy and Trabeculectomy with Application of Mitomycin C in managing Primary Congenital Glaucoma. Pak J Ophthalmol.

[22] Mullaney PB, Selleck C, Al-Awad A, Al-Mesfer S, Zwaan J (1999). Combined trabeculotomy and trabeculectomy as an initial procedure in uncomplicated congenital glaucoma. Arch Ophthalmol.

[23] Al-Hazmi A, Awad A, Zwaan J, Al-Mesfer SA, Al-Jadaan I, Al-Mohammed A (2005). Correlation between surgical success rate and severity of congenital glaucoma. Br J Ophthalmol.

[24] Sood D, Rathore A, Sood I, Singh G, Sood NN (2018). Long-term outcome of combined trabeculotomy-trabeculectomy by a single surgeon in patients with primary congenital glaucoma. Eye (Lond).

